# Laboratory Studies about Microplastic Aging and Its Effects on the Adsorption of Chlorpyrifos

**DOI:** 10.3390/polym15163468

**Published:** 2023-08-19

**Authors:** Sílvia D. Martinho, Vírgínia Cruz Fernandes, Sónia A. Figueiredo, Rui Vilarinho, J. Agostinho Moreira, Cristina Delerue-Matos

**Affiliations:** 1 REQUIMTE/LAQV—ISEP, Polytechnic of Porto, Rua Dr. António Bernandino de Almeida 431, 4249-015 Porto, Portugal; silvia.martinho@graq.isep.ipp.pt (S.D.M.); cmm@isep.ipp.pt (C.D.-M.); 2 Department of Chemistry and Biochemistry, Faculty of Sciences of the Porto University, Rua do Campo Alegre s/n, 4169-007 Porto, Portugal; 3 Department of Physics and Astronomy, Faculty of Sciences of the Porto University, Rua do Campo Alegre s/n, 4169-007 Porto, Portugal; ruivilarinhosilva@gmail.com (R.V.); jamoreir@fc.up.pt (J.A.M.); 4 IFIMUP—Institute of Physics for Advanced Materials, Nanotechnology and Photonics, Faculty of Sciences of the Porto University, Rua do Campo Alegre 687, 4169-007 Porto, Portugal

**Keywords:** aged microplastic, pristine microplastic, river water ecosystems, seawater ecosystems, weathering effects

## Abstract

The constant change in microplastics (MP) due to exposure to environmental conditions leads to physical and chemical changes that enhance their ability to transport other pollutants, increasing the concern about their widespread presence in the environment. This work aimed to simulate the aging process of six MP (polyamide 6, unplasticized polyvinyl chloride, low-density polyethylene, polystyrene, polyethylene-co-vinyl acetate, polypropylene) in freshwater and seawater ecosystems at laboratory scale and evaluate its effects through optical microscope observation, Fourier transform infrared spectroscopy-Attenuated Total Reflectance (FTIR-ATR), Raman spectroscopy, and thermal gravimetric analysis (TGA). Through a combined experimental study of aged MP, the degradation by UV interaction was evidenced by the appearance of new infrared bands in the FTIR spectra assigned to ketones and hydroxyl groups. While Raman analysis and microscope images reveal the appearance of pores, wrinkles, and roughness in the MP surfaces. Variations in the temperature of the maximum weight loss of the MP were observed in the TGA analysis. The adsorption of chlorpyrifos (CPF), a common pesticide widely used in agriculture, by the pristine and aged MP was also studied. The highest affinity for CPF was observed for pristine LDPE and the lowest for PP. The batch adsorption studies revealed an increase in adsorption capacity as a consequence of the aging process for both MP. These results proved that the weathering effects caused changes in the behavior of MP, namely in the interaction with other pollutants.

## 1. Introduction

Plastics and their key properties such as light weight, strength, durability, and low cost make them one of the most useful materials in modern life, with numerous applications in the everyday life of the population [1,2]. The use of plastics in several industries is still common due to their unique properties that are difficult to achieve with other materials. They are widely used for packaging, construction, electrical and electronics, automotive, and agriculture [3,4,5]. Large-scale production of plastics, for single and long-term use, results in a huge plastic waste burden on terrestrial and aquatic ecosystems [4,6]. According to the literature, the global production of plastics is about 300 million tons per year, with a recycling rate of about 32.5%, so, the rest of the plastic is disposed of in landfills [7]. The researchers estimated that 10% of plastic production ends up in the oceans, where they undergo various transformations under weathering conditions due to mechanical, radiation, chemical and biological effects [8]. The degradation of plastic wastes takes place in the environment, resulting in small plastic particles with a diameter of less than 5 mm, known as microplastics (MP) [6,8,9,10,11]. In the past, MP pollution was thought to be greater in marine ecosystems; however, in 2019, the terrestrial environment was found to be 4 to 23 times more contaminated [12,13].

Microplastics (MP) may have two different sources: directly from industry (e.g., cosmetics, pharmaceutical) which is called the primary source, or MP can be the result of fragmentation and degradation of large plastics, which is considered a secondary source [9,11]. These tiny particles are reported as highly persistent and ubiquitous throughout the environment and may be found in different forms, such as granules, fibers, fragments, and pellets [8,9,14]. Their presence is an increasing concern, as studies have already confirmed the MP presence in liquid water (e.g., freshwater, groundwater, rainwater, bottled and tap water) [15,16,17,18], snow and ice [16,18,19], soil, sediments and sands [15,16,17,20], terrestrial and aquatic biota [21,22]. Their detection was also confirmed in food products [23,24] and, more recently, in biological samples (e.g., blood, placenta, lung tissues, hand and facial skin, saliva, colon) [25,26,27,28]. These results clearly ascertain that MP are ingested by living organisms, such as seabirds [8,9,10,29], fish [8,9,10,29], turtles [10], whales [10], crabs [10], prawns [30], mussels [8,30] invertebrates [21,29,31], earthworms [12,21,32] and can also alter plant root traits [33]. Several organisms ingest MP in confusion with food, which causes several damages in these organisms, such as inhibition of food intake and reproduction [8,34,35], oxidative stress effect [12,34], and energy metabolism disturbance [34]. Consequently, humans are also ingesting MP through the food chain; this poses a higher risk to human health [21,36,37]. Moreover, MP can sorb and transfer other pollutants, such as pesticides [8,9,30], pharmaceuticals [38,39], metals [12,13,34], polychlorinated biphenyls (PCBs) [8,9,10,12], and polycyclic aromatic hydrocarbons (PAHs), which enhances their harmful effects [8,9,10,12].

Characteristics, such as the polymer structure, particle size, large specific surface area, and strong hydrophobicity may favor the interaction of MPs with different pollutants [17,35,40,41,42]. While MP are spread in the environment, multiple weathering conditions cause their aging, such as UV radiation, chemical oxidation, biodegradation and mechanical stress [8,9,13]. These environmental processes are directly associated with the aging process of MP and it is reported that these alterations can pose a big threat to the environment compared to pristine MP. Aged MP can present significant differences in their properties when compared with pristine MP, like significant visual changes in the color, surface area and roughness, through the appearance of cracks, bulges, wrinkles, oxidized particles, pores and in the color of the particles [10,16,42,43]. Chemical changes in MP have been ascertained to structural modifications, which are identified through changes in the thermal stability of MP, including shifts in melting and glass transition temperatures. Moreover, the aging process can result in the emergence of new functional groups (such as –HO, C=O, COOH, C=C, C–Cl) on the surface of microplastics, as reported in previous studies [44,45,46]. These changes in MP properties have been found to facilitate interactions with various types of contaminants (e.g., pesticides, pharmaceuticals, metals, PCBs, PAHs), increasing the potential risks associated with their presence in the environment.

Brennecke et al. [47] and Wang et al. [48] demonstrated that aged polyethylene terephthalate (PET) and aged polyvinyl chloride (PVC), had higher adsorption efficiency for two types of heavy metals, copper (Cu^2+^) and zinc (Zn^2+^), compared to pristine MP. Changes in roughness and specific surface area after the aging process of MP are reported in the adsorption process of the pesticide atrazine (ATZ), highlighting the major adsorption process on the aged PE [30]. Chen et al. [49] studied the adsorption capacity of PVC and expanded polystyrene (EPS), after submitting the MP to an irradiation process, which led to an increase in the sorption capacity of bisphenol (biphenol F, A, B and AP) [49]. Understanding the impact that MP can have and study their interactions with different pollutants that are disperse in the environment is crucial for recognizing the various sectors that need behavioral changes to minimize the negative impacts associated [10,34]. Comprehending the extension of MP contamination and the identification of the key areas is essential to promote measures to mitigate and reduce the adverse effects caused by these ubiquitous pollutants [12,15].

The use of plastic mulches, application of soil conditioners and irrigation of fields with plastic tubes are mentioned as possible inputs of MP into agricultural fields [50]. The application of sewage sludge for soil fertilization can also be considered an important route of MP contamination of fields, as it is estimated that 125–850 t of MP per million habitants are added to European soil each year by farmlands [22]. Beyond the concern of MP dispersion, their aging process due to weathering effects favors the interactions with other contaminants in the ecosystems, such as pesticides, which are common soil pollutants [8,9,30]. Lan et al. compared the adsorption behavior of pristine and aged PE soil films testing four different pesticides (carbendazim, diflubenzuron, malathion, and difenoconazole) and concluded that aged MP presented a higher capacity to adsorb the pesticides than the pristine ones, becoming a vector for the dispersion of these pollutants [51]. This concern is supported by the presence of a “cocktail of pesticides residues” in the Ebro River Bassin (Mediterranean zone) where Ccanccapa et al. reported the presence of 50 pesticides: chlorpyrifos, diazinon and carbendazim were the most frequently detected in water samples during the study (2010–2011) [52].

Various pesticides are widely used in agriculture to control pests with the main objective of increasing the production yield. Chlorpyrifos (CPF) has been used since 1960 and is widely used in domestic and agricultural pest control [53]. This organophosphorus insecticide inhibits the enzyme acetylcholinesterase and impairs the transmission of nerve impulses in the central nervous system, resulting in acute and chronic toxicity to non-target wildlife [17,54]. CPF has been classified as a priority substance in the European Union Water Framework Directive, and quality standards have been set for this compound to protect the aquatic environment and human health, and its use has been regulated in EU countries and onboard [17,54]. Due to its extensive use, its presence has been reported in food and water, and in different environments, such as coastal areas, and it has also been found adsorbed by plastic waste in coastal areas [55,56,57].

This work aims at exploring the effects of the aging process in various MPs using a simple set-up to simulate mechanical stress and photooxidation. In this experiment, polyamide 6 (PA6), unplasticized polyvinyl chloride (UPVC), low-density polyethylene (LDPE), polystyrene (PS), polyethylene-co-vinyl acetate (EVA) and polypropylene (PP) were submitted to artificial weathering experiments for two months, and they were analyzed by several analytical techniques to evaluate the aging degree. The influence of the aqueous matrix, sea and river water was also tested to understand its impact in a real environment. Preliminary tests of CPF adsorption were performed for comparison of the behavior of pristine and aged MP. Preliminary kinetic studies were carried out for two systems, PP (river and seawater—PP aging system) and LDPE (river and seawater–LDPE aging system) and which represent MP with low and high affinity to CPF, respectively.

## 2. Materials and Methods

### 2.1. Reagents and Solutions

Analytical-standard CPF with high purity (≥98%) was obtained from Sigma Aldrich-Merck (Darmstadt, Germany). Chromatography-grade *n*-hexane was purchased from Merck (Darmstadt, Germany). Analytical-grade ethyl acetate from Dasit Group (Val de Reuil, France) and dichloromethane from Sigma Aldrich-Merck (Darmstadt, Germany). Stock solutions of the pesticide were prepared in *n*-hexane (10,000 μg L^−1^) and were kept in darkness and refrigerated at 4 °C. Working-standard solutions were prepared by appropriate dilution of the stock solutions in *n*-hexane. For the aging procedure, a certified artificial sandy soil from MIBAL—Minas de Barqueiros S.A. (Apúlia, Portugal) was used, which had approximately 90% silica content.

Low-density polyethylene (LDPE) colorless powder with a particle size lower than 300 μm, polyethylene-co-vinyl acetate (EVA) yellow granules with a particle size between 3 and 5 mm, unplasticized polyvinyl chloride (UPVC) colorless powder with a particle size lower than 250 μm, polyamide 6 (PA6) colorless spherical with an average size between 15 and 20 μm, polystyrene (PS) colorless granules with a diameter of 3 to 5 mm, and polypropylene (PP) colorless granules with a diameter of 5 mm were supplied by Goodfellow (Hamburg, Germany).

### 2.2. Water Samples Used in the Aging Process Systems

River water samples, with a pH of 7.63 and conductivity of 6.80 mS/cm, from the Douro River, Portugal (GPS 41.1414, −8.6518) and seawater samples, with a pH of 8.06 and conductivity of 39.8 mS/cm, from Canide Sul Beach, Portugal (GPS 41.1129, −8.6637)) were collected in glass bottles in October of 2022. Both samples were filtered using a nylon filter (Membrane Solutions, Auburn, WA, USA) with a pore size of 0.45 μm to remove suspended solids. The water samples were collected from large water bodies, such as the Douro River and the Atlantic Ocean; therefore, the seasonality influence is reduced and is considered representative of the river and seawater environments, namely with different salinities.

### 2.3. Procedure

#### 2.3.1. MP Aging Process Laboratory Setup

A volume of 100 mL of each filtered water was placed in a glass cylinder into which 25 g of silica sand had previously been placed. A set of 12 cylinders was used for the aging process of the 6 MP and named according to the type of MP and water involved in the process (e.g., Seawater–LDPE aging system, River water–LDPE aging system). Each cylinder was provided with 300 mg of MP. All assays were continuously aerated through a silicone tube equipped with an air pump (Sera Air 550R Plus) to promote agitation. All tubes were placed in an intermediate position in the cylinder to ensure the movement of all particles, from sand to MP, and to simulate the mechanical abrasion of MP. The whole experiment was performed under six cool white fluorescent lamps (84 lm/W) (Philips Master, TL-D 36W/865, Warsaw, Poland) to simulate the continuous daylight irradiation of MP during 1440 h (2 months). Evaporation was considered and the water level was replenished twice a week, to ensure the same conditions during the test period. Every 15 days, the positions of all cylinders were changed to ensure the same conditions for the different MP and waters throughout the experiment. At the end of the experiment, samples were filtered with a 0.22 μm glass filter to extract the MP from each water-MP aging system.

#### 2.3.2. MP Characterization: FTIR, Raman and Thermogravimetry Analysis

Infrared measurements were made using a Fourier Transform Infrared (FTIR) spectrometer (PerkinElmer, Waltham, MA, USA) equipped with a single reflection attenuated Total Reflectance (ATR) diamond, operation in the 4000–600 cm^−1^ mid-IR region and 32 scans/samples. All spectra were corrected for light reflectance penetration and baseline displacement.

Micro-Raman spectra were recorded at room temperature, using a Renishaw InVia Qontor spectrometer, equipped with an optical microscope. This study was performed using 633 nm of a He-Ne laser as excitation. The maximum laser power used was 100 mW, which has been found to be suitable for the measurements without heating the samples. The microscope is equipped with a 50× long working distance lens and the scattered light was registered in the 3500–100 cm^−1^ spectral range. The spectral resolution is better than 2 cm^−1^.

Thermal gravimetric analysis (TGA) from Netzsch (STA449) was used, equipped with a Pt/Rh crucible (diameter of 6.8 mm, volume 85 μL) with a lid. The sample was heated from 25 to 600 °C with a temperature slope of 10 K min^−1^ in a streaming nitrogen atmosphere (purge: 50 mL min^−1^) to suppress oxidation. The gradient and measurement started at 25 °C. An empty Pt/Rh crucible served as a reference test. Integration of the TGA peaks was performed using Proteus Thermal Analysis Software from Netzsch.

#### 2.3.3. Optimization of the Liquid-Liquid Extraction of Chlorpyrifos from Water-MP System

An aliquot of 1 mL of each of the standard solutions of CPF (500 μg L^−1^) in distilled water was placed in a glass flask (Linex—Vilabo, Marinha Grande, Portugal) of 20 mL and an aliquot of 1 mL of the extraction solvent was added to the flask in a 1:1 ratio. It was carried out in duplicate. Three immiscible water solvents were tested (*n*-hexane, dichloromethane, and ethyl acetate) to find the optimal extraction conditions. The mixture was vigorously agitated on a vortex (VWR—Analog Vortex mixer, Radnor, PA, USA) for 1 min. The formation of fine droplets during the stirring facilitated the contact between chlorpyrifos and the solvent, and after this step, the solution rested for 15 min. During this time, two different layers formed spontaneously, and a volume of 500 μL of the organic phase was collected with a micropipette (VWR, Radnor, PA, USA) and filtered into a new glass flask using a PTFE filter of 0.22 μm (BGB Analytik, Böckten, Switzerland). After filtration, the extract was injected into a gas chromatograph (GC) with a flame photometric detector (FPD). Extractions were performed in duplicate and the GC analysis was in triplicate, as described in Section 2.3.4.

The extraction efficiency was evaluated in order to choose the best solvent. The efficiency of each solvent was determined by calculating the extraction efficiency (R%), according to Equation (1):

(1) R%=CextC0×100,

where C_ext_ (μg L^−1^) and C_0_ (μg L^−1^) are the extracted concentration and initial concentration of chlorpyrifos, respectively.

#### 2.3.4. GC Analysis

The GC-FPD (GC-2010, Shimadzu, Kyoto, Japan) was used to determine the chlorpyrifos concentration. A sample volume of 1.4 μL was injected in splitless mode, using an injector temperature of 250 °C. The starting temperature of the column oven was 100 °C and was maintained for 1 min. The temperature was increased to 170 °C at a rate of 20 °C min^−1^ and held for 1 min, then increased to 210 °C at a rate of 4 °C min^−1^ and held for 1 min, and finally increased to 300 °C, at a rate of 15 °C min^−1^, and kept for 5 min. Helium (Nippon Gases, Maia, Portugal), at 1 mL/min with a linear velocity of 25.4 cm/s was used as a carrier gas. A Zebron-5MS column from Phenomenex (Madrid, Spain), with dimensions 30 m × 0.25 mm i.d. × 0.25 μm was used. The calibration curve was performed in the CFP concentration range of 40 and 800 μg L^−1^.

#### 2.3.5. Preliminary Batch Adsorption Studies for CPF/Pristine MP

A volume of 50 mL of aqueous chlorpyrifos solution (150 μg L^−1^) was put in contact with 50.0 mg weight of all the six pristine MP (LDPE, PA6, UPVC, PP, PS, EVA) (corresponding to a concentration of 1 g L^−1^), in a glass Erlenmeyer flask. The initial samples were collected before adding the MP. Control samples (without MP) were run during the experimental period (96 h). The glass Erlenmeyer flasks were shaken continuously by an orbital (Orbital shaker AO-400, Busen) at 110 rpm, and aliquots of 1.0 mL were collected after 1 min of vigorous shaking in the vortex (VWR—Analog Vortex mixer, Radnor, PA, USA). An aliquot of 1.0 mL of each system was collected and filtered using a PTFE filter (0.22 μm pore size diameter) and a liquid-liquid extraction with *n*-hexane followed by the CPF determination was performed as described in Section 2.3.3 and Section 2.3.4, respectively. The removal efficiency was calculated using the same formula as the recovery efficiency (Equation (1)) to compare each MP behavior. The adsorption capacity of the MP was calculated according to Equation (2) for the kinetic studies:

(2) $$q_{t}=\frac{\left(C_{0}-C_{t}\right)\times V}{m}$$

where C_0_ and C_t_ are the initial and concentration solution in a determined period (μg L^−1^), V is the volume of CPF solution at that time t and m (g) is the MP mass.

#### 2.3.6. Batch Adsorption Experiments

Preliminary kinetic batch adsorption experiments were performed to study the equilibrium time and adsorption rate of CPF by PP (river and seawater–PP aging system) and LDPE (river and seawater–LDPE aging system) systems, which represent the studied MP with the highest and lowest affinity to CPF, respectively. An amount of 50.0 mg of MP was weighed into a glass Erlenmeyer flask of 100 mL. A 50.0 mL volume of aqueous solution of CPF (around 150 μg·L^−1^), prepared with distilled water, was added to the MP and submitted to continuous orbital stirring at 110 rpm (Orbital shaker AO-400, Busen) for 96 h. Initial samples were collected in duplicate to measure the initial concentration of CPF. Aliquots of 1.0 mL were collected at defined time intervals, and after 30 s of vortex agitation, were filtered with a PTFE filter of 0.22 μm in a glass flask. Then liquid-liquid extraction of CPF (Section 2.3.3) was performed. Blanks (solution without MP) were performed in parallel to the kinetic study in duplicate. A schematic diagram of the adsorption experiments is supplied in the supplementary materials (Figure S1). All experiments were carried out at room temperature (20 °C) and the same procedure of extraction and analysis was followed (Section 2.3.3 and Section 2.3.4). The adsorption capacity of the MP was calculated according to Equation (2). Kinetic parameters were estimated using non-linear regression to fit three different models: Pseudo-1st order [58], Pseudo-2nd order [59] and Elovich’s models [60] using the software program Origin Pro 8.5.

## 3. Results and Discussion

### 3.1. Water-MP Aging Systems

The water-MP aging systems were subjected to different aging factors for 2 months. Pristine and aged MP were characterized concerning the effects on their structure and composition. This study evaluated the aging degree of MP through FTIR-ATR, Raman spectroscopy and thermogravimetric (TGA) to identify the physical and chemical changes.

#### 3.1.1. Characterization by Optical Microscopy

**Figure 1:**
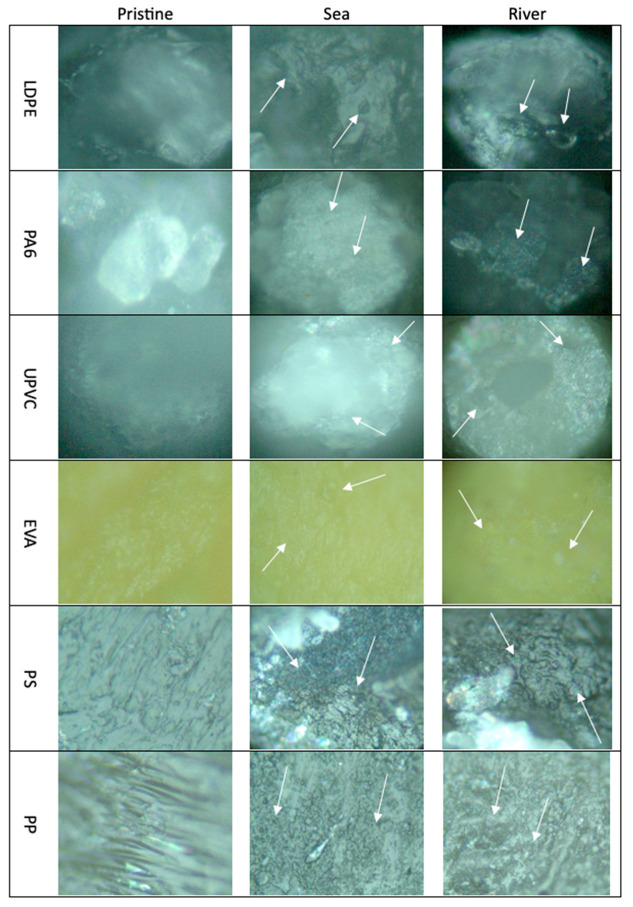
Optical microscope images of the 6 MP pristine (**left**), seawater-MP (**middle**) and river water-MP aging systems (**right**), highlighting with arrows the most important physical changes.

Pristine and aged MP were observed using an optical microscope (Figure 1). In this study, the images captured were used to evaluate the physical changes that have occurred, comparing the pristine MP (left side) with the aged MP-sea (middle) and the aged MP-river (right side). The arrows (Figure 1) highlight the most visible surface changes. The smallest MP, like LDPE, PA6 and UPVC were more difficult to observe; however, compared with the pristine forms of these MP, it was possible to observe that the surfaces were completely changed due to the appearance of several pores and the roughness of the surface has increased too. These aging changes were more evident in the PS and PP surface images, where it is possible to observe a significant change in the porosity of the material and an increase in the roughness. In EVA images, despite being possible to observe some alterations in the porosity of the MP, it was difficult to capture them due to the yellow color of the MP.

Similar results have been reported by other authors showing that when PS and PVC were submitted to a UV-accelerated aging process, the formation of small wrinkles during the aging process, as cracks in the PS surface, is observed in the SEM images [61]. You et al. studied (SEM) the artificial photoaging of PE, identifying the appearance of wrinkles and roughness on the yellow surface of the yellow aged-PE when compared with the white pristine PE with a smooth flat surface [62]. Several studies reported significant physical changes in the aged MP when compared with the pristine, such as changes in color, and the appearance of cracks, bulges, wrinkles, pores, and MP surfaces [62,63,64,65,66]. Optical or fluorescence microscopy and electron microscopy (Scanning electron microscopy—SEM or Transmission electron microscopy—TEM) are other commonly reported techniques used to study the physical changes of MP [35,46,67].

#### 3.1.2. Characterization by FTIR Analysis

**Figure 2:**
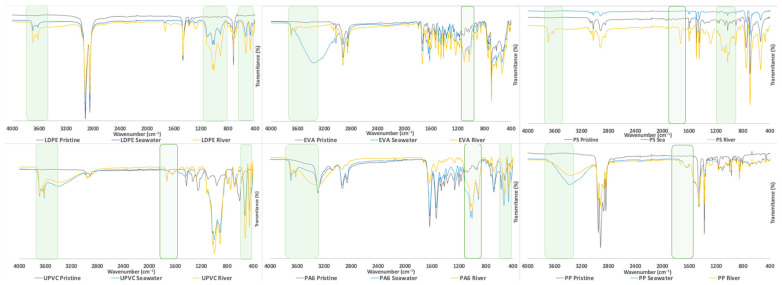
FTIR spectra of the 6 MP pristine, seawater-MP and river water-MP aging systems, highlighting the significant spectra changes with green squares.

FTIR analysis is a useful technique to identify changes in the highly polar chemical bonds, which occur as a consequence of the aging process. The FTIR spectra of the pristine MP (grey dash) and aged MP in sea (blue dash) and river (yellow dash) waters are shown in Figure 2, where the wavenumber ranges with more significant changes were highlighted in green. Also, the expanded version of the spectra is presented in the Supplementary materials (Figure S2). It was possible to observe that new peaks appear in the spectra of the aged MP. The most prominent spectral changes are observed in the ranges 3800–3300, 1200–800 and 600–400 cm^−1^ for all MP, which is evidence of chemical changes due to the aging process, as will be discussed [68].

As reported in the current literature, the appearance of new functional groups, such as ketones (1720 cm^−1^), carboxylic acids (1713 cm^−1^), esters (1735 cm^−1^), lactones (1780 cm^−1^) and double bonds (1640 cm^−1^) is related to the aging process [63,69]. Bands represented in the hydroxyl vibration frequencies, with a maximum at 3420 cm^−1^ and a sharp absorption band at 3550 cm^−1^ represent the formation of monomeric hydroperoxides and hydrogen-bonded alcohols and hydroperoxides (3550 cm^−1^ and 3420 cm^−1^, respectively) [69]. The formation of new bands between 1900 and 1500 cm^−1^ indicates the formation of products after a thermo and photooxidation process. According to the obtained results, it is possible to observe in the FTIR spectra of LDPE, UPVC and PP for both aging systems (sea and river) and PS-river, the formation of the new band around 1725 cm^−1^ which is characteristic of ketones. Ketones are considered one of the main products that result from thermal oxidation, so it is expected that this appearance shows the changes in this MP after aging. However, ketones were not identified in the spectra of PA6 and EVA, for both aging systems and seawater–PS, which may be related to some experimental constraints that occurred. For seawater–EVA, river water–EVA and seawater–PS aging systems an increase in turbidity was observed, despite being the same river water for all aging systems. This different behavior was observed despite the constant position change of the assays to ensure an equal distribution of the light. The occurrence of turbidity decreases light penetration, preventing the occurrence of photooxidation. During the aging process, PA6 particles with size 15 to 20 μm tended to adhere to the walls of the glass cylinder and EVA particles with size 3 to 5 mm tended to be trapped in the sand. To avoid this behavior, when the water was refilled, during the aging process, it was passed through the glass walls of the cylinder to release the amount of PA6 that was attached. Also, aeration helps EVA particles to be released. These different behaviors could lead to different exposure to the artificial weather effects and protect the particles from suffering aging processes such as photooxidation.

The emergence of additional peaks in the FTIR spectra within the range of 3750–3000 cm^−1^ can be attributed to the presence of hydroxyl groups [68]. These hydroxyl groups are likely a result of exposure to UVA irradiation, which is known to accelerate the aging and degradation of the MP material being analyzed [70]. Furthermore, the increased contact with oxygen may also contribute to the formation of these hydroxyl groups. The appearance of these new bands in the FTIR spectra provides valuable insights into the chemical changes occurring in the material due to UVA irradiation and oxygen exposure [70]. The appearance of the aforementioned bands in the FTIR spectra has been used to prove the aging of MP [46,71,72,73,74]. Andrade et al. studied the aging process of PP, PS and PA6 using a homemade low-cost weathering system after 2 months of experiment and observed that the MP presented an increase in the number of peaks in FTIR spectra in the carbonyl and hydroperoxides bands. New peaks were detected at 3360–3240 cm^−1^, corresponding to the hydroxyl groups, at 1640 cm^−1^ representing the double bonds formed or C=O groups and at 1100 cm^−1^ that correspond to the C–O bonds [68].

In the study of Wu et al. the PP was submitted to a photooxidation process simulated at a laboratory scale using three different types of water: ultrapure, estuary and seawater. After the 20 days, the aged-PP were analyzed by FTIR and it was possible to observe the appearance of new functional groups: at 1653 cm^−1^ (O–H stretching), at 1712 cm^−1^ (carboxylic acid), at 1735 cm^−1^ (esters) and 1780 cm^−1^ (lactones) [75].

#### 3.1.3. Characterization by Raman

The Raman technique provides information about color and texture changes through the analysis of small areas (2–4 μm^2^), which is complementary to the data concerning chemical changes given by FTIR analysis results, and where a wider area is analyzed. The appearance of fluorescent compounds, identified as a background change, is understood as an indication of changes caused by MP aging [76].

**Figure 3:**
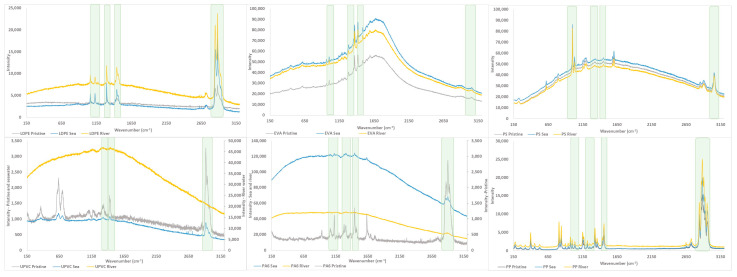
Raman spectra of the 6 MP pristine, seawater-MP and river water-MP aging systems, highlighting the significant spectra changes with green squares.

The Raman spectra, shown in Figure 3, were acquired for all the pristine, seawater-MP aging systems and river water-MP aging systems. Also, the expanded version of the spectra is presented in the Supplementary materials (Figure S3). It is possible to observe that most of the analyzed MP present the same characteristic bands, which identify the polymers that compose the MP (LDPE, PP, PS, EVA), both in pristine and aged form, but for UPVC and PA6 is not possible to observe these bands in the river water-MP aging systems due to the background increase that superposes to the Raman signal, suggesting the occurrence of changes due to aging. However, most of these bands present a redshift, which can be associated with the changes promoted by the aging process, highlighted in green in Figure 3. Furthermore, there are very significant changes in the spectra, relative to changes in background intensities, which can be indicative of chemical structure changes, due to fluorescence associated with the presence of organic molecules.

In Figure 3 is possible to observe an increase in the spectra background in the river water-MP aging systems for the MP that present smaller particle sizes, such as UPVC and PA6. For the small particles of MP (UPVC and PA6), the spectra were masked by the increase in the background noise, making the Raman analysis impossible, despite the attempts of careful separation (filtration and washing) of the MP from the water and the sand, in several filters was possible to observe some contamination. This experimental constraint reveals the urgency of exploring an improvement in the separation technique for the freshwater samples, once it has a significant impact on the results. In contrast to the seawater samples, where flotation of MP was favored due to the salinity, mostly due to the presence of sodium chloride (NaCl). Also, as previously exposed, the river water environment has favored the development of biofilms.

The variation on the wavenumber was noticed in the bands at 1062 cm^−1^ and 1127 cm^−1^ corresponding to the C–C stretching modes, at 1296 cm^−1^ assigned to the CH_2_ twisting modes of the crystallinity chains, at 1427 cm^−1^ that corresponds to the bending and wagging modes of the crystallinity chain and at 1438 cm^−1^ that is assigned to the bending mode of the amorphous trans chain. Some researchers suggest that these changes in the spectra shifts could correspond to the physicochemical changes induced by long-term solar radiation exposure and external stress [77,78].

Raman spectroscopy is considered a good technique to be used in the MP analysis due to the specific information on high molecular-weight polymers and the easy identification of MP. However, it is also mentioned that there are some limitations like the fluorescence of sample compounds that may interfere with the MP identification; a purification step of the samples is recommended to reduce fluorescence [35].

In this study, despite the easy identification of MP, the purpose was to understand the impact of the aging process on the MP through the Raman spectra analysis, but some changes were not quite evident. Even for the identification of some polymers, different researchers try to complement the analysis of Raman with the ATR-FTIR, like in Kappler et al.’s study [74]. For the analysis of sediment samples, the authors used the FTIR and Raman spectroscopy. Although one of the particles (white fragmented particle) was not identified as an MP using the Raman analysis and was considered as an inorganic particle of TiO_2_, when the same sample was analyzed by FTIR, with a database, it was identified as PS. Researchers use both results to conclude that could be a polymeric matrix of acrylic resin with an inorganic white pigment of TiO_2_, therefore, both analyses were needed [74]. Imhof et al., also used the results of both techniques, once the Raman microspectroscopy was not able to detect polymer in paints [79].

#### 3.1.4. Characterization by TGA

**Figure 4:**
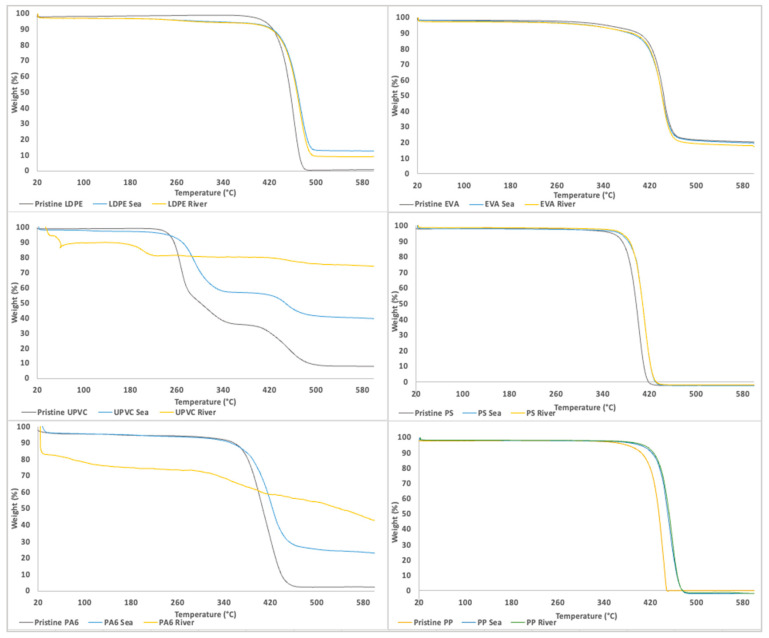
TGA results of the 6 MP pristine (**left**), seawater-MP aging systems (**middle**) and river water-MP aging systems (**right**).

TGA analysis of pristine and aged MP was performed and represented in Figure 4.

Based on the experimental findings, weight loss was observed in various materials at different temperatures. Pristine LDPE experienced a weight loss of 98.4% at 485 °C, while the aged LDPE samples from sea and river lost 86.9% and 86.8% of their weight, respectively, at 497 °C. In the study of Dalai et al., the highest loss of LDPE weight was observed at 476 °C [80].

Pristine UPVC exhibited a weight loss of 90.7% at 503 °C, whereas the aged UPVC samples from the sea lost 56.4% of their weight at 483 °C. Pristine PA6 lost 91.8% of its weight at 458 °C, and the aged PA6 samples from the sea experienced a weight loss of 71.2% at 463 °C, in accordance with the results of Zhao et al., which present a temperature of 450 °C for the maximum weight lost of PA6 [81].

The weight loss of pristine and aged EVA samples from the sea and river occurred at 473 °C, with weight losses of 78.2%, 88.4%, and 88.2%, respectively. Also, Dalai et al. showed that the highest decomposition of EVA was observed at 470.1 °C [80].

Pristine PS underwent complete weight loss (100%) at 423 °C, while the aged PS samples from the sea and river lost 99.6% and 99.3% of their weight, respectively, at 432 °C. Miandad et al. studied the pristine PS degradation and achieved a decomposition of 91% at 450 °C [82,83].

As in the previous analyses, some constraints were found in the UPVC and PA6 aged-river due to the difficulty in the separation of the MP. The small amount of MP that was collected was not enough to execute the TGA experiments, being unable to detect the TGA evolution of this MP.

The obtained results provide valuable insights into the thermal stability and degradation behavior of the tested materials. The observed weight loss at specific temperatures indicates the susceptibility of the materials to thermal decomposition. The differences in weight loss between pristine and aged samples suggest that aging and exposure to environmental factors, such as sea and river conditions, can influence the thermal degradation of the materials. These findings contribute to a better understanding of the thermal properties and performance of LDPE, UPVC, PA6, EVA, PS, and PP materials under different aging conditions.

### 3.2. Liquid-Liquid Extraction of Chlorpyrifos with Different Solvents

Before the adsorption studies, it was crucial to ensure a good extraction efficiency of the CPF from the aqueous solution to allow its analysis by GC. A liquid-liquid extraction procedure was tested by using three solvents, *n*-hexane, ethyl acetate and dichloromethane, selected due to their immiscibility in water and high solubility of CPF. Table 1 presents the results of the extraction efficiency obtained for the three organic solvents tested. Despite the solubility of CPF in the three solvents being similar, higher than 400 g·L^−1^ [84], it is possible to observe in Table 1 that *n*-hexane presents the best recovery ratio with 99% for the CPF, followed by ethyl acetate and at last dichloromethane with 38% of recovery ratio. CPF solubility in water is relatively low, around 1.2 mg·L^−1^, and it was not influenced by the pH range of this work (between 7 and 9).

According to the results, *n*-hexane and ethyl acetate were considered efficient solvents for CPF extraction from aqueous solutions. The selection of *n*-hexane was related to the obtention of the highest recovery (99% ± 5%), good chromatographic behavior in GC analysis and minimization of experimental steps, by eliminating the solvent exchange step.

The calibration curve was performed for CPF analysis by GC-FPD. The coefficient of determination (R^2^) was 0.9931 demonstrating good linearity. All samples were collected in duplicate, analyzed in triplicate and subjected to validation with a maximum relative standard deviation of 15%.

### 3.3. Batch Adsorption Studies for CPF/Pristine MP Systems

**Figure 5:**
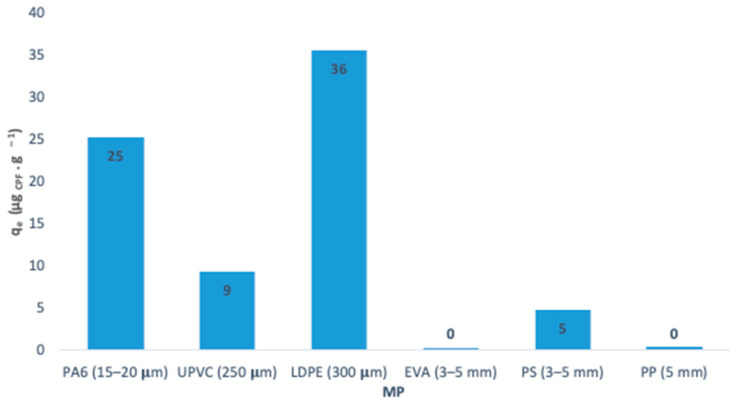
Removal of CPF onto six MP after 96 h of contact.

The six pristine MP (PA6, UPVC, LDPE, EVA, PS and PP) were tested to evaluate their affinity with the CPF. A concentration range between 1.00 and 1.25 g·L^−1^ of MP was put in contact with CPF-contaminated water (150 μg·L^−1^) for 96 h, to guarantee that equilibrium was reached. In Figure 5 it is possible to observe the percentage removal and adsorption capacities of CPF for the six pristine MP studied.

This preliminary study of the six MP in contact with the aqueous solution of CPF shows that LDPE and PA6 have a higher affinity to interact with this pesticide. The particle size plays an important role in the adsorption of different contaminants by the MP because, in low porosity materials, like MP, the specific surface area available for adsorption increases when the particle size decreases. Particle size directly affects the adsorption process, its reduction causes an increase in the specific area and amount of available adsorption sites, increasing the adsorption capacity [85]. Also, the chemical composition of LDPE and PA6 may contribute to a higher affinity, when compared with UPVC which presents a low particle size too.

Concha-Granã et al. studied the adsorption capacity of PA6 in contact with CPF-contaminated water and concluded that 80% removal by adsorption was achieved, which can be related to the polar groups present in the PA6 that are allowed to retain hydrophilic compounds, but this reduces the ability to retain hydrophobic substances. The adsorption on PA6 was quite variable, and the best adsorption capacity of CPF was observed at 4 °C due to the decrease in CPF water solubility as a consequence of the temperature decrease, which favors the adsorption to the plastic [86].

LDPE has the highest removal percentage of CPF, meaning that presents a higher adsorption capacity for CPF. In the Garrido et al. study at least 70% of the CPF was adsorbed into polyethylene (PE, with a maximum particle size of 22 μm) after 2 h of incubation [87]. CPF has a high octanol-water partition coefficient, log K_ow_ = 4.66, which might explain the high affinity to the polyethylene particles [88]. Different authors considered that PE when compared with other plastics, presents the highest adsorption capacity of MP [89,90].

EVA, PS and PP did not show significant adsorption of CPF (Figure 5), as expected given the influence of the particle size. Due to their size in the scale of millimeters, between 3 and 5 mm, it is predicted a decrease in the specific surface area available for adsorption is reduced when compared with the other MP.

Although the UPVC size could favor the adsorption process of CPF, a low affinity to CPF was observed. Other properties affect the adsorption process, such as the crystallinity of the material, which can have an important effect on the partitioning of hydrophobic organic compounds. According to previous classifications, PVC is considered an amorphous MP [91]. The amorphous transition from a rubbery phase to a glassy phase when the temperature drops below the glass transition temperature (Tg). PVC is a glassy plastic, due to the Tg being above ambient temperature [91]. Researchers explored the influence of the rubbery and glassy MP and concluded that rubbery MP may have higher mobility, diffusivity and accessibility than the glassy MP. The free volume in the internal cavities is revealed to be higher in the rubbery than the glassy MP, so it is expected a lower adsorption capacity of glassy MP, like UPVC, in the presence of other contaminants [92].

### 3.4. Adsorption Kinetic Studies

Preliminary kinetic studies were performed to understand the behavior of MP when in contact with the CPF in aqueous solutions. According to the results of Section 3.3, LDPE represents the most critical situation, followed by PA6. However, due to the difficulty in the separation step of PA6 faced in the aging studies, the kinetic experiments will be performed only for LDPE, both for seawater and river water–LDPE aging systems. Studies with the seawater and river water–PP aging systems were also performed to understand if the aging process could cause an increase in CPF adsorption.

**Figure 6:**
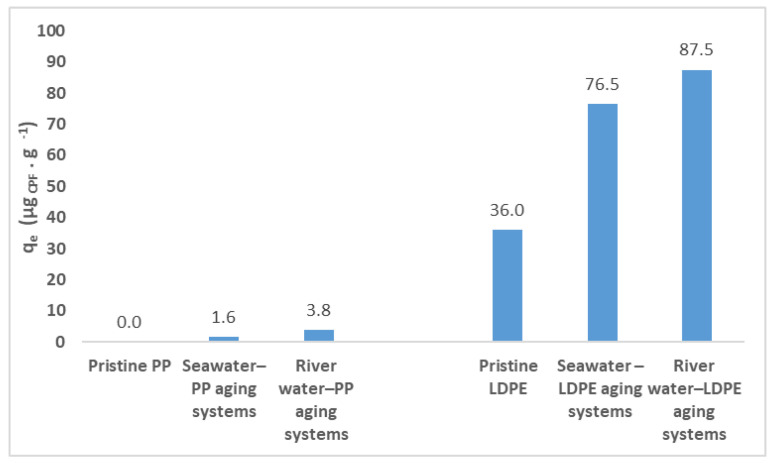
Comparison of experimental adsorption capacities of pristine and aged (seawater and river water) for PP and LDPE.

In Figure 6 it is possible to observe the comparison of adsorption capacities of pristine and aged (seawater and river water) for PP and LDPE, after reaching the equilibrium, where an increase of the adsorption capacity was observed as a consequence of the aging process.

**Figure 7:**
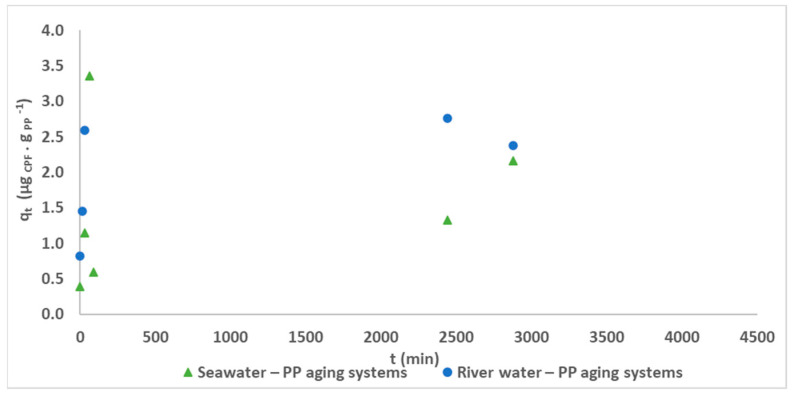
Evolution of the CPF concentration over time in the presence of seawater/river water–PP aging systems.

The results of the kinetic studies for the PP aging systems and for seawater and river water–LDPE aging systems are presented in Figure 7. It is possible to observe that the equilibrium was reached after 24 h of contact. Although in the preliminary study, pristine PP did not present a significant affinity to the adsorption of CPF, the several changes observed as a consequence of the aging process have caused an increase in the adsorption behavior due to the physicochemical changes observed: the formation of new functional groups in the FTIR spectra (such as ketones at 1725 cm^−1^ and the hydroxyl stretching vibration, corresponding to the –HO stretching, at 3750–3000 cm^−1)^ and surface changes, namely the appearance of pores and roughness (identified in the Raman images). During the kinetic experiments, the PP aged in seawater presented a lower affinity to CPF than the PP aged in river water. This different behavior may be explained by the development of microorganisms (biofilm formation), which is more favorable in river waters than in sea waters, due to their salinity. The formation of biofilms on the surface of the MP particles may explain the increase in the adsorption of the CPF by the river water aged PP. The biofilm formation increases the hydrophobicity due to the attack of the weaker components of the MP, which favors the adsorption of hydrophobic contaminants, such as CPF [44]. Considering the low affinity observed in the PP aging systems, no further treatment of the results was performed.

**Figure 8:**
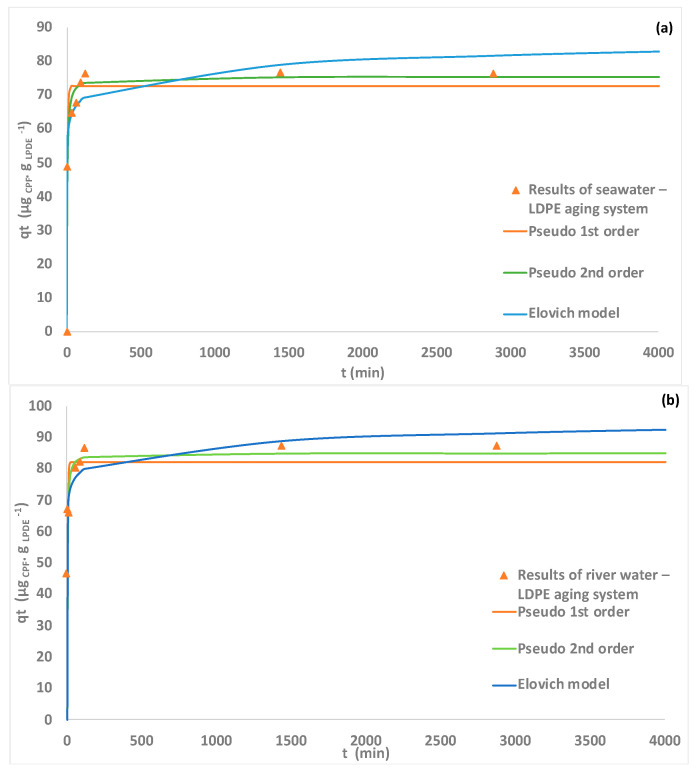
Kinetic experimental data with fitted models: pseudo-1st order model (orange dash), pseudo-2nd order (green dash) and Elovich’s model (blue dash), for adsorption of CPF onto seawater–LDPE aging system (a) and river-water LDPE aging systems (b).

LDPE was the MP that showed the highest affinity to adsorb CPF with 79% removal, corresponding to an adsorption capacity to the pristine LDPE of 36 µg _CPF_ . g _LDPE_ ^−1^ (Figure 5). The results of the kinetic studies for the seawater and river water–LDPE aging systems are shown in Figure 8.

According to the results, it is possible to observe that after 60 min of contact, the concentration of the pesticide was reduced to half and then slowly decreased until reaching equilibrium, after 24 h of contact. The pseudo-1st order, pseudo-2nd order and Elovich models were fitted to the experimental results (Figure 8). The models can represent the kinetic results and their parameters and are presented in Table 2. For the seawater–LDPE aging system, the highest coefficient of correlation and the lowest residual sum of squares were obtained for the pseudo-2nd order model (0.991) and for the river water–LDPE aging system were obtained for the Elovich’s model (0.979), although the adjusted models are considered statistically equivalent to represent the kinetic results. The equilibrium adsorption capacities estimated by the different models are in accordance with the experimental values reported. The highest adsorption capacity was observed for the river water–LDPE aging system (87.5—experimental; 82 µg _CPF_ . g _LDPE_ ^−1^—pseudo-1st order model; 85 µg _CPF_ . g _LDPE_ ^−1^—pseudo-2nd order model), followed by the seawater aging system (76.5 — experimental; 73 µg _CPF_ . g_LDPE_ ^−1^ — pseudo-1st order model; 75 µg _CPF_ . g _LDPE_ ^−1^ — pseudo-2nd order model) and the pristine LDPE (36 µg _CPF_ . g _LDPE_ ^−1^—experimental). The use of river water in the experiments promotes the formation of biofilms around the MP particles, increasing their hydrophobicity and favoring the adsorption of CPF, as observed for the corresponding PP aging system.

The LDPE/CPF interactions had been explored by researchers with a focus on the ecotoxicity of the microalgae and marine copepod. Garrido et al. assessed the toxicity of MP and CPF to the microalgae *Isochrysis galbana*, exposing a constant concentration of MP (5 mg·L^−1^) to a CPF solution with a range of 0–3 mg·L^−1^. After an incubation of 2 h, it was possible to observe that 80% of CPF was sorbed onto the MP surface [87]. The combined effects of CPF with PE were explored on the survival, fecundity, feeding and egg viability of *Acartia tonsa*, a calanoid copepod in Bellas et al.’s study. During this experiment, it was possible to observe that CPF sorbed onto PE showed higher toxicity than CPF in solution, reinforcing the hazardous role of MP as a vector for the transport of other pollutants [54].

## 4. Conclusions

The impact of MP has been an increasing concern due to their wide dispersion through the environment. Moreover, MP are in constant change due to the different environmental conditions, such as ultraviolet radiation, thermal degradation, oxidation reactions and biodegradation, that continuously modify the properties of MP and enhance their ability to transport pollutants into various environments.

In this work, six different types of MP were submitted to laboratory conditions to simulate the aging process in water systems, using river and sea waters as matrixes. The characterization of pristine and aged MP was performed through optical microscope observation, FTIR-ATR, Raman and TG analysis to evaluate the changes in the different MP properties. Significant changes were observed in the optical microscope images, making it possible to observe the appearance of pores and the roughness in the surface of the different tested MP. According to the FTIR results, the formation of new bands was associated with the formation of products after the thermal and photooxidation process, namely ketones, which are considered one of the main products of thermal oxidation. These observations were complemented by Raman analysis, where spectra background changes were identified due to the presence of fluorescent compounds, which are associated with the effects of the aging process. TG results showed differences in the weight loss behavior between the pristine and aged MP at specific temperatures, indicating the susceptibility of these MP to thermal decomposition. The differences between pristine and aged MP samples suggest that aging, due to exposure to environmental factors influences the degradation of the polymers.

The affinity of CPF, a commonly used pesticide, onto pristine MP was tested. The LDPE presented the highest adsorption capacity (36 µg _CPF_ . g _LDPE_ ^−1^). Preliminary kinetic studies were performed for the MP with the highest and lowest affinity, respectively, LDPE and PP, both for the river and seawater aging systems, to evaluate the effects of aging on the adsorption capacity of CPF. An increase in the adsorption capacity was verified due to the aging process, which was more evident in the river water aging system due to the biofilm formation around the MP particles, which increases the hydrophobicity of MP due to the attack of its weaker components, favoring the adsorption of hydrophobic contaminants, such as CPF. This behavior change was consistent with the chemical, structural and morphological changes identified in the characterization performed in the pristine and aged MP, both in sea and river water simulated environments.

## Figures and Tables

**Table 1:** Extraction efficiency in aqueous samples (average of duplicate experiments).

Organic Solvent	C_0_ (μg·L^−1^)	Recovery Ratio (%)	RSD (%)
*n*-hexane	150	99	5
Ethyl acetate		91	12
Dichloromethane		38	8

**Table 2:** Parameters calculated with the different kinetic models for the adsorption of CPF/Water-MP aging systems.

Models	Parameters	Seawater–LDPE Aging System	River Water–LDPE Aging System
Pseudo-1st order	k_1_ (min^−1^)	0.22 ± 0.04	0.31 ± 0.09
	q_e_ (µg _CPF_. g _LPDE_^-1^)	72.70 ± 1.89	82.15 ± 3.06
	R^2^	0.973	0.947
	Residual sum of squares	128.94	324.57
Pseudo-2nd order	k_2_ (g _LPDE_ . µg _CPF_ ^−1^ . min^−1^)	0.0044 ± 7.78 × 10^−4^	0.006 ± 2.12 × 10^−3^
	q_e_ (µg _CPF_. g _LPDE_^-1^)	75.39 ± 1.29	85.03 ± 2.36
	R^2^	0.991	0.975
	Residual sum of squares	44.82	148.98
Elovich	α (µg . g _LPDE_ ^−1^ . min^−1^)	1.83 × 10^6^ ± 8.27 × 10^6^	1.79 × 10^8^ ± 8.96 × 10^8^
	Β (µg . g _LPDE_ ^−1^)	0.258 ± 6.88 × 10^−2^	0.286 ± 6.58 × 10^−2^
	R^2^	0.962	0.979
	Residual sum of squares	183.24	127.65

## Data Availability

Not applicable.

## Supplementary Materials

The following supporting information can be downloaded at: https://www.mdpi.com/article/10.3390/polym15163468/s1, Figure S1: Schematic diagram of adsorption experiments; Figure S2: Expanded FTIR spectra of the 6 MP, pristine, seawater and river water aging systems; Figure S3: Expanded Raman spectra of the 6 MP, pristine, seawater and river water aging systems.
