# Supplementary material for: Laboratory Studies about Microplastic Aging and Its Effects on the Adsorption of Chlorpyrifos
Source: Polymers (Basel). 2023 Aug 19;15(16):3468. doi: 10.3390/polym15163468 (PMC10459960; doi:10.3390/polym15163468)
Supplement: Supplementary file 1 [file polymers-15-03468-s001.zip › polymers-2539578-supplementary.pdf]

polymers

MDPI

Article

Laboratory studies about microplastic aging and its effects on the adsorption of chlorpyrifos

Sílvia D. Martinho [1,2], Vírgínia Cruz Fernandes [1,*], Sónia A. Figueiredo[1,*], Rui Vilarinho[3,4], J. Agostinho Moreira[3,4] and Cristina Delerue-Matos[1]

1 REQUIMTE/LAQV – ISEP, Polytechnic of Porto, Rua Dr. António Bernandino de Almeida 431, 4249-015, Porto, Portugal
2 Departamento de Química, Faculdade de Ciências, Universidade do Porto, rua do Campo Alegre s/n, 4169–007 Porto, Portugal.
3 Department of Physics and Astronomy, Faculty of Sciences of the Porto University, Porto, Portugal
4 IFIMUP – Institute of Physics for Advanced Materials, Nanotechnology and Photonics, Faculty of Sciences of the University of Porto, Porto, Portugal
* Correspondence: vir@isep.ipp.pt/saf@isep.ipp.pt; Tel.: +351 228340500

Supplementary Materials:

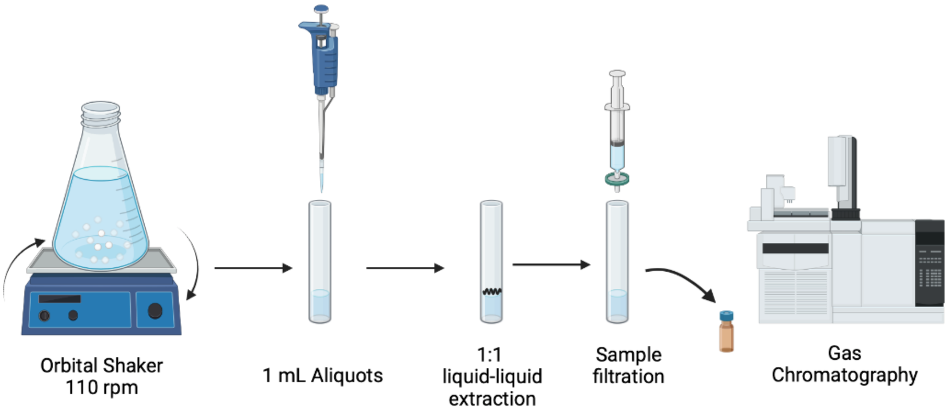

Figure S1. Schematic diagram of adsorption experiments.

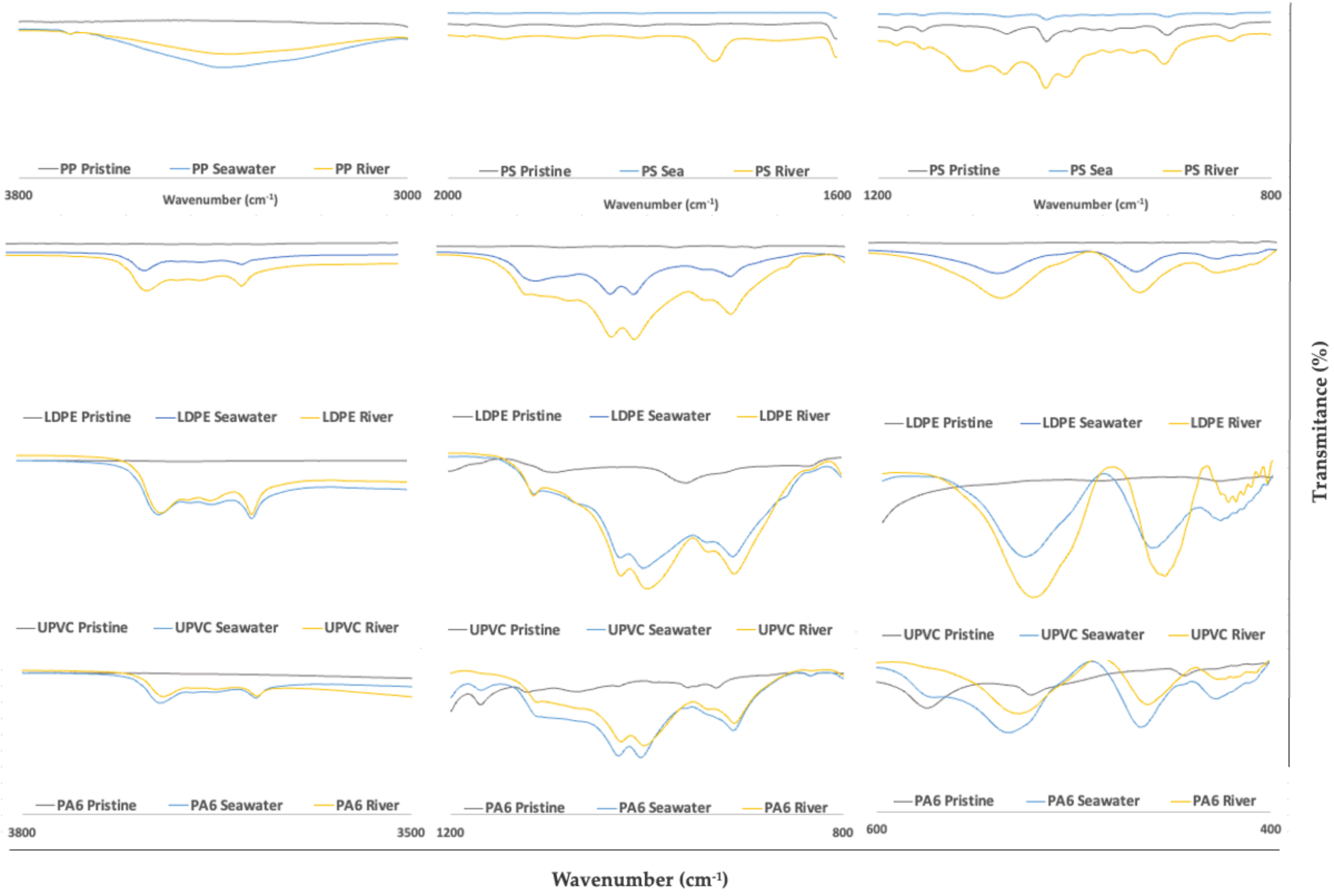

Figure S2. Expanded FTIR spectra of the 6 MP, pristine, seawater and river water aging systems.

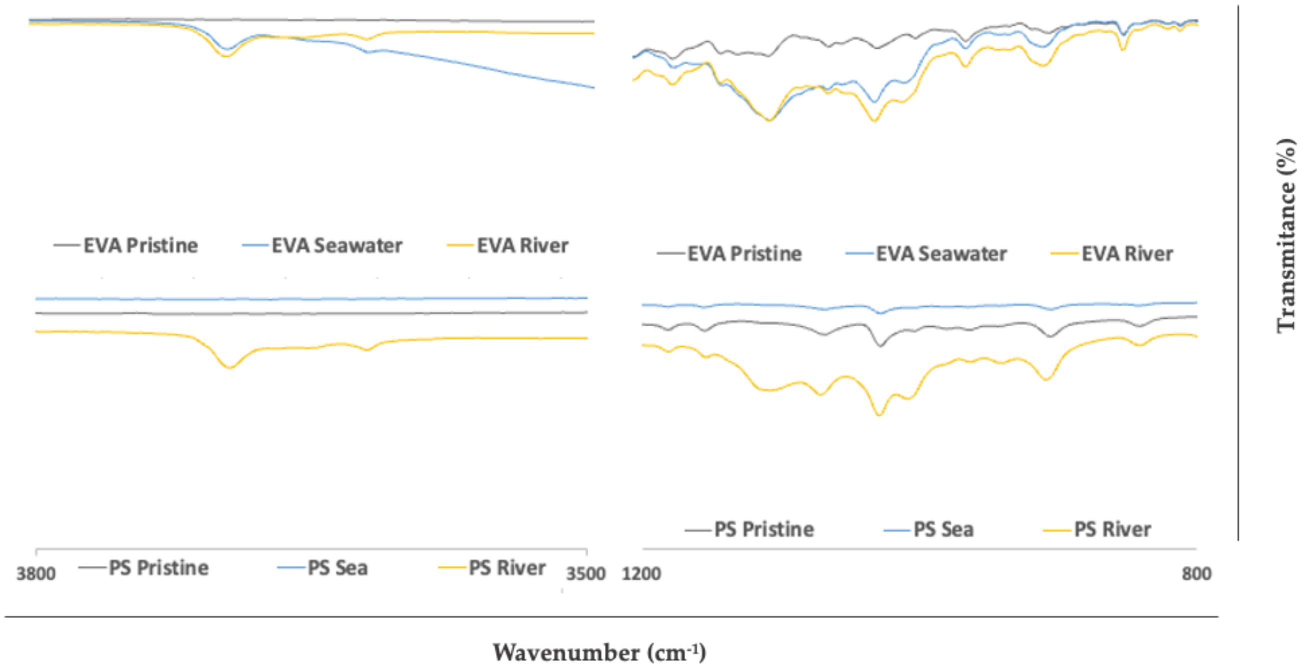

Figure S2. Expanded FTIR spectra of the 6 MP, pristine, seawater and river water aging systems (Cont.).

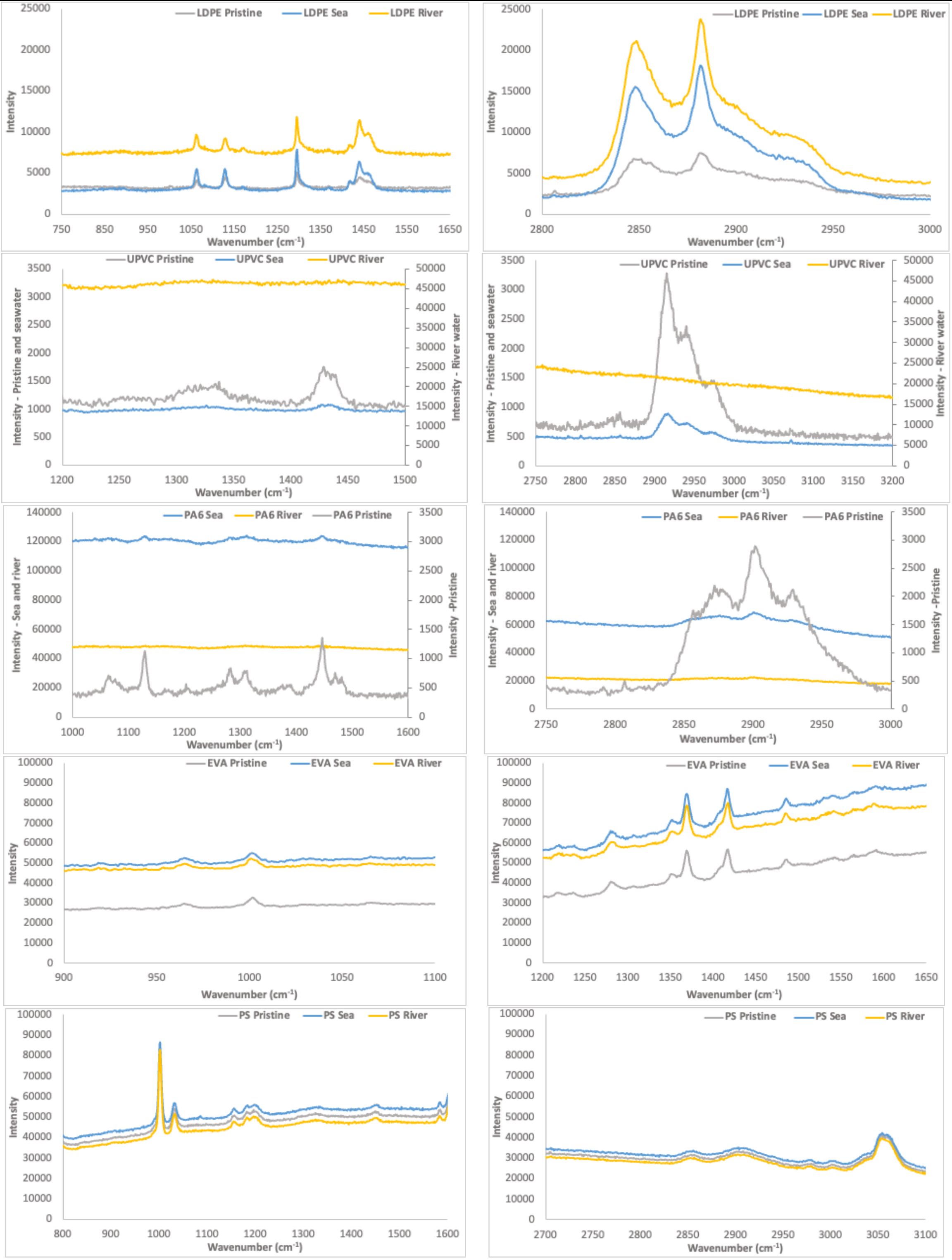

Figure S3. Expanded Raman spectra of the 6 MP, pristine, seawater and river water aging systems.

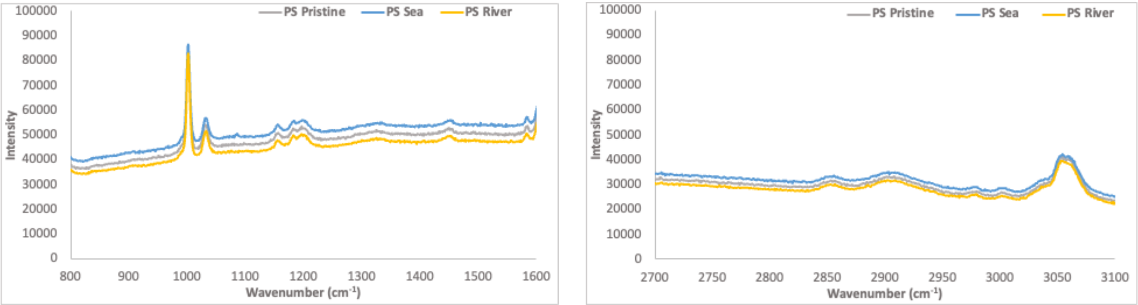

Figure S3. Expanded Raman spectra of the 6 MP, pristine, seawater and river water aging systems (Cont.).
